# Rehabilitation and Pain Management in Klippel-Feil Syndrome Associated With Caroli's Disease, Scoliosis, and Aphasia

**DOI:** 10.7759/cureus.94535

**Published:** 2025-10-14

**Authors:** Amine Achraf Majit, Araj Aymane, Ahmed Amine El Oumri

**Affiliations:** 1 Physical Medicine and Rehabilitation, Faculty of Medicine and Pharmacy, Mohammed I University, Oujda, MAR; 2 Physical Medicine and Rehabilitation, Mohammed VI University Hospital, Oujda, MAR

**Keywords:** caroli’s disease, congenital spine deformity, kfs, klippel-feil syndrome, motor aphasia, pain management, scoliosis, spine rehabilitation

## Abstract

Klippel-Feil syndrome (KFS) is often underdiagnosed. Medical imaging studies suggest that its incidence may be higher than currently recognized. Although surgical treatment has been extensively studied, non-surgical approaches, such as physiotherapy, rehabilitation, and pain management, receive comparatively less attention. We report the case of an 18-year-old girl being treated for a malformation syndrome consisting of a rare condition, Caroli’s disease with aphasia, who was referred for movement limitations associated with scoliosis and cervicobrachial neuralgia. The main objective of this article is to present a case of malformation syndrome associated with KFS, Caroli’s disease, and scoliosis in an aphasic patient, as well as the difficulty of medical management, mainly rehabilitation and pain management outside of surgery.

## Introduction

Klippel-Feil syndrome (KFS) is a rare congenital disorder, first described in 1912 by Maurice Klippel and André Feil, characterized by fusion of two or more cervical vertebrae [1]. Although early studies estimated the incidence at 1 in 42,000 births, with 60% of cases occurring in women [1,2], more recent imaging-based studies suggest a prevalence as high as 1 in 172 births, likely due to prior underdiagnosis [3]. KFS is frequently associated with skeletal, renal, auditory, and cardiac anomalies, including scoliosis, Sprengel deformity, and congenital heart malformations [4,5]. Caroli’s disease is a rare congenital disorder characterized by cystic dilatation of the intrahepatic bile ducts, yet no cases have been reported describing its association with KFS.

KFS is listed in the Online Mendelian Inheritance in Man database as being of sporadic autosomal dominant inheritance with reduced penetrance and variable expression [6]. Almost all cases of this syndrome occur sporadically; nevertheless, close evaluation of the immediate family is recommended [7]. KFS can lead to variable neurological symptoms such as radiculopathy and myelopathy often result from instability of adjacent vertebral segments or spinal canal stenosis. The prognosis depends on the type and extent of the fusion: extensive or craniocervical fusions typically present earlier and more severely, while limited fusions may remain asymptomatic and are often discovered incidentally [5].

## Case presentation

**Figure 1:**
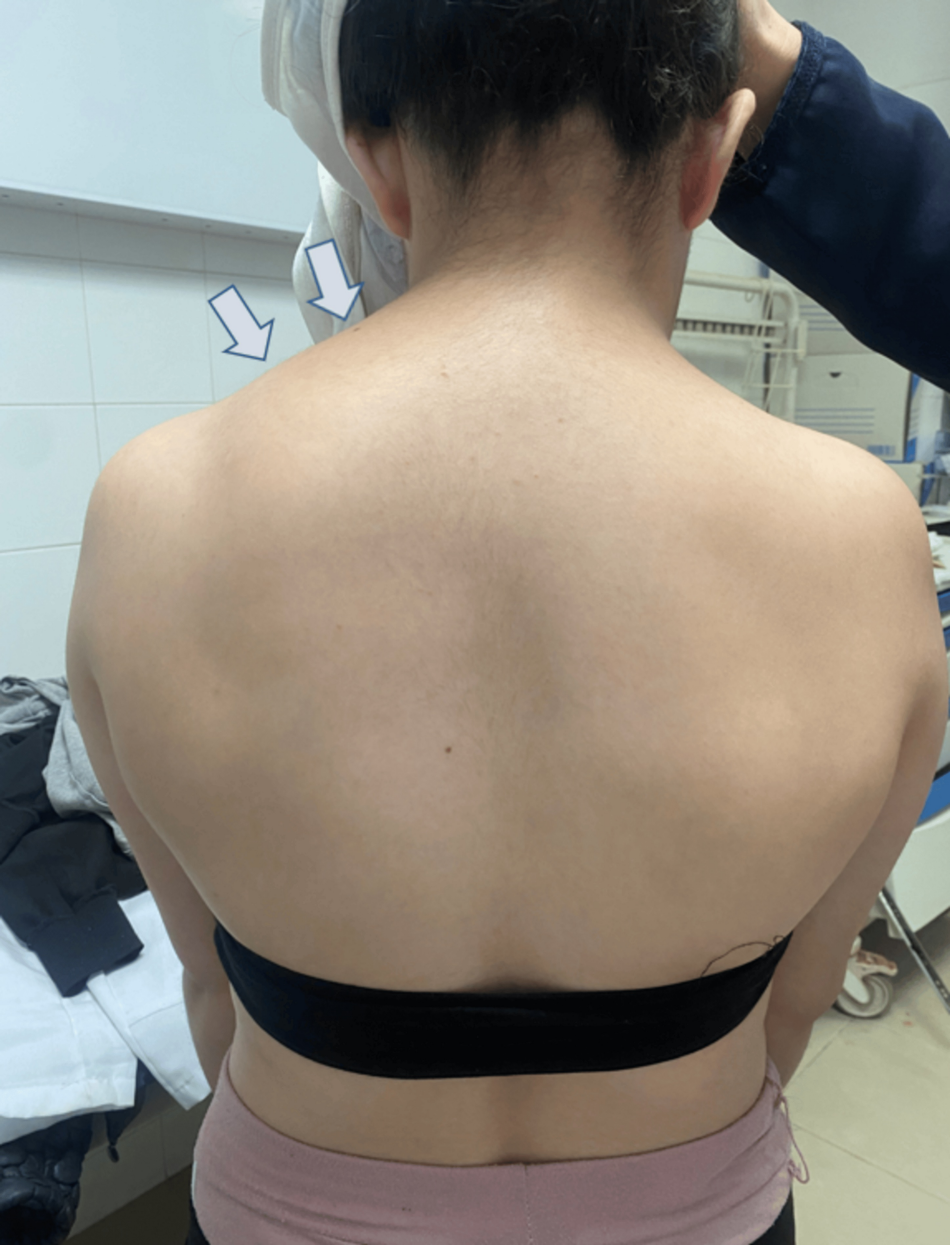
Clinical aspect showing short neck, low hairline, increased thoracic kyphosis, and Sprengel deformity (arrows).

This is an 18-year-old patient who was referred for limitation of movement with cervical spine deformity. The patient complained of cervicobrachial neuralgia, particularly on the right, with pain estimated at 4/10 on the visual analogue scale; no motor deficit was reported by the patient. The medical history, which was challenging to obtain due to the patient's aphasia, revealed an appendectomy performed at the age of 10 years, a diagnosis of Caroli’s disease at the age of 11 years, and no history of cervical trauma. On clinical examination, the patient presented with a short neck, a low posterior hairline, well-developed (webbed) trapezius muscles, and Sprengel deformity (Figure 1). An antalgic trunk deviation was observed. Cervical range of motion (RoM) was limited in both active and passive mobilization in all directions. All tendon reflexes were normal, and no neurological deficits were noted.

**Figure 2:**
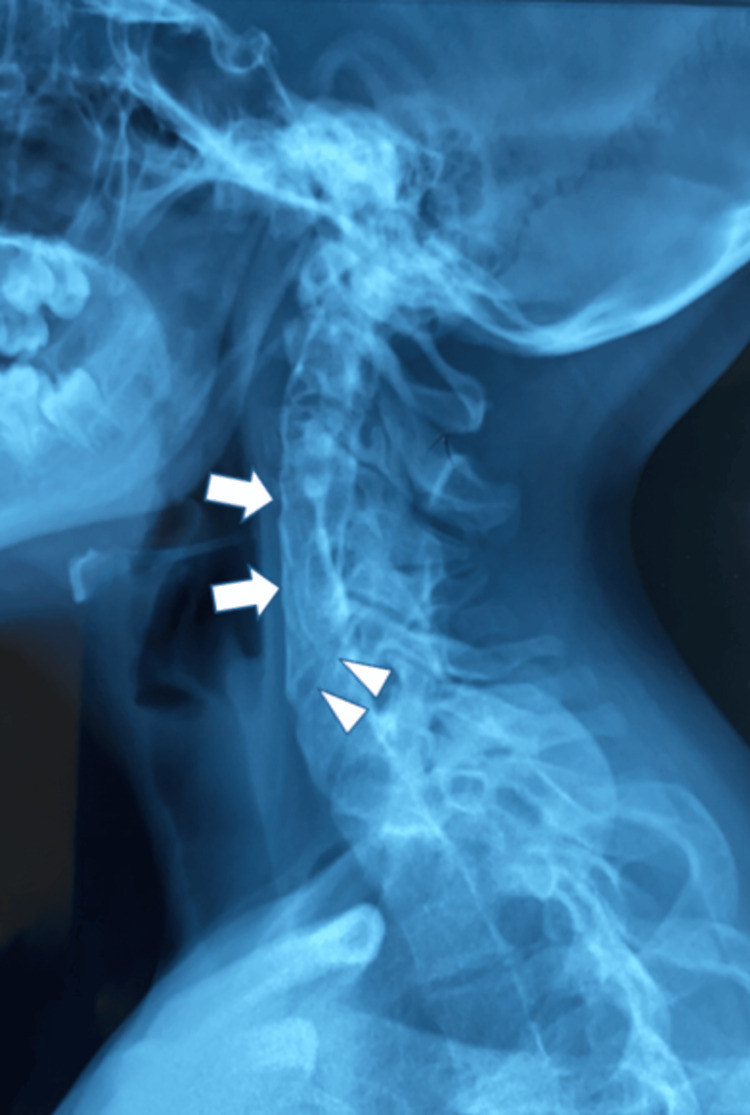
Left lateral radiograph shows total fusion of the C3-C4-C5 vertebral bodies (arrows) with partial posterior fusion of the C5-C6 vertebral bodies (arrowheads).

**Figure 3:**
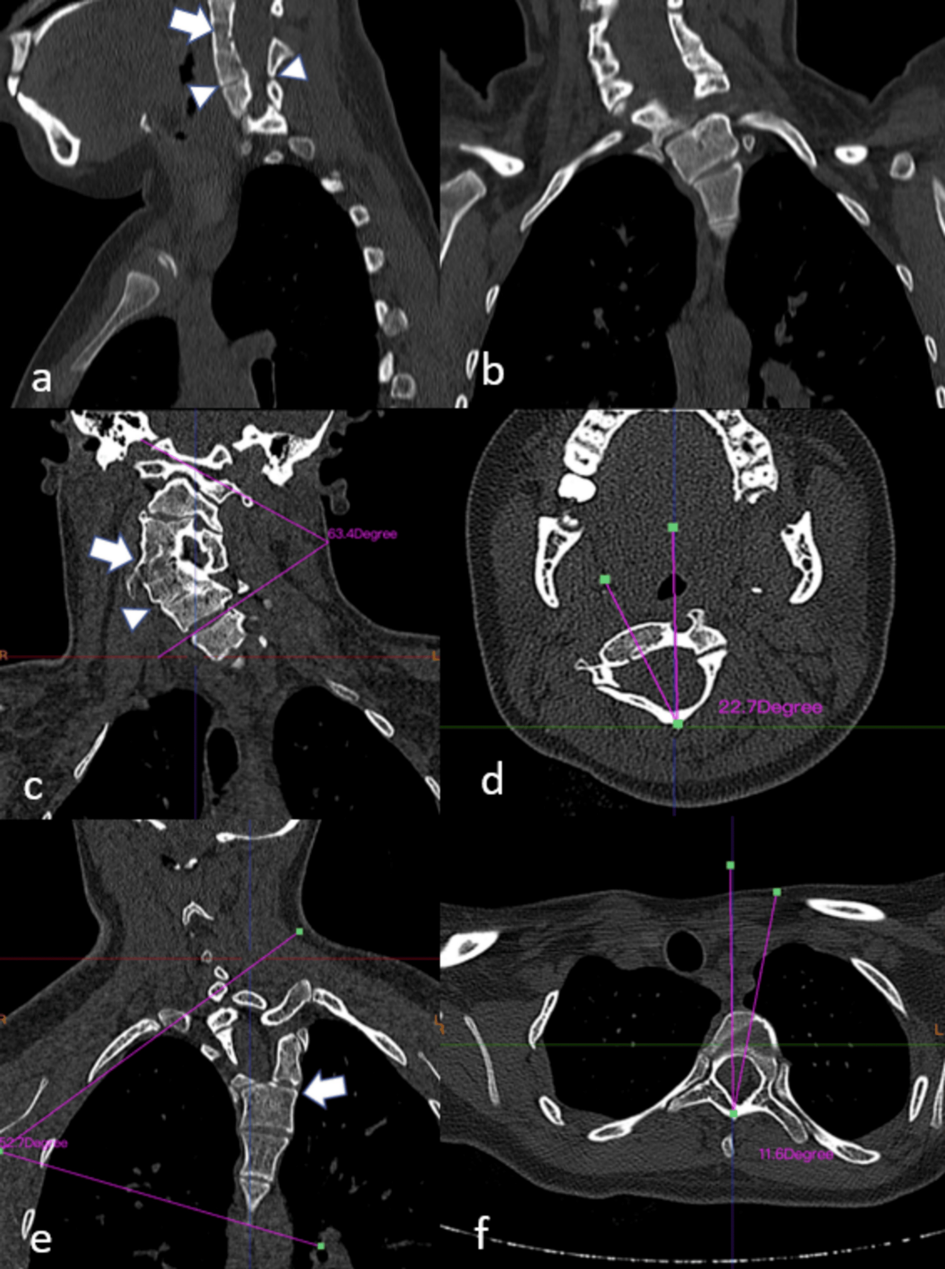
Cervical computed tomography (CT) scan showing total fusion of the C3-C4-C5 vertebral bodies with partial posterior fusion of the C5-C6 vertebral bodies. Sagittal view (a), coronal view (b), cervical scoliosis (arrow) with a Cobb angle of 63.4°, partial posterior fusion of the C5-C6 vertebral bodies (arrowhead) (c), apical cervical vertebra rotation in the axial plane of 22.7° (d), thoracic scoliosis (arrow) with a Cobb angle of 52.7° (e), and apical dorsal vertebra rotation in the axial plane of 11.6°(f).

**Figure 4:**
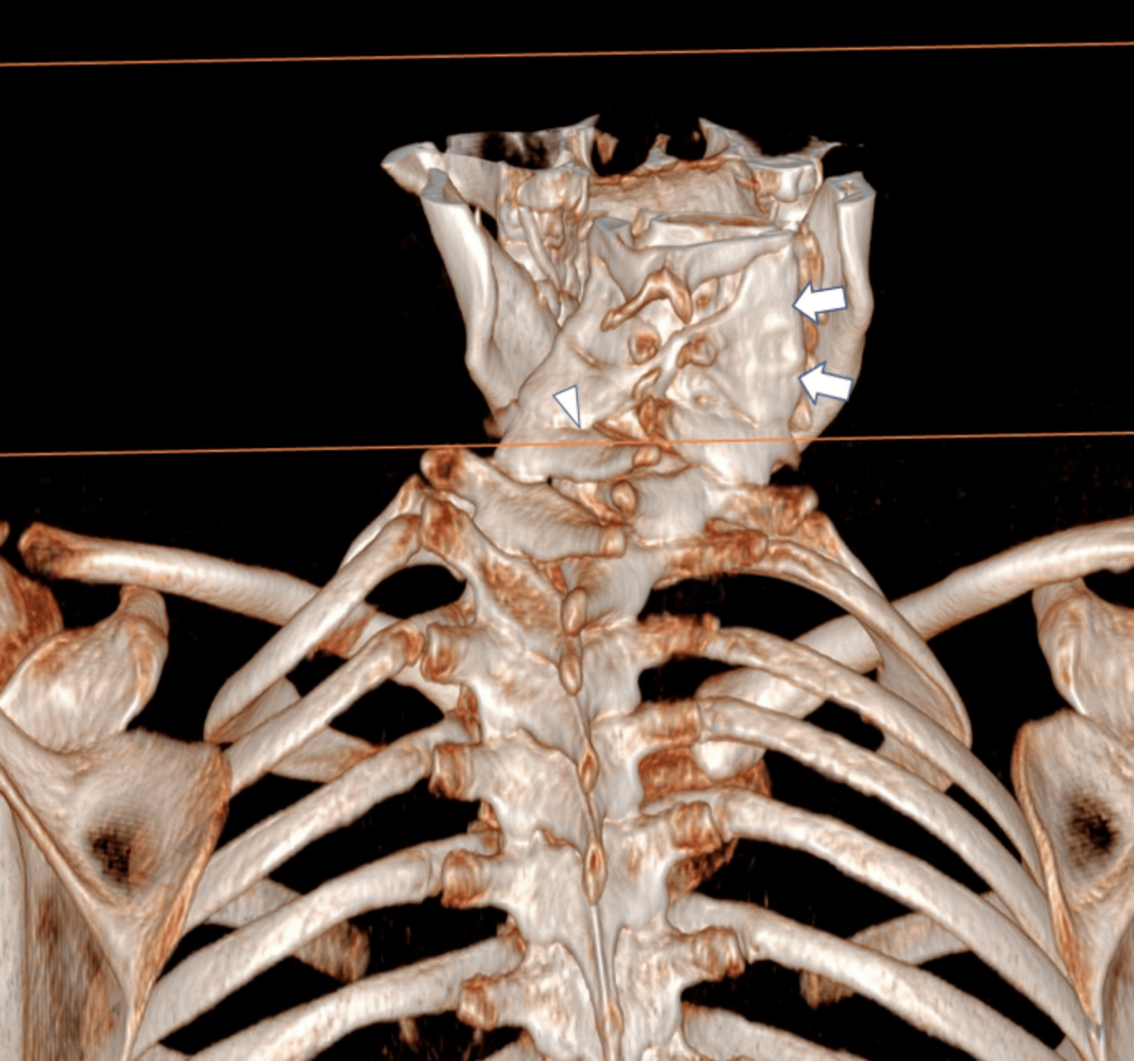
Volume rendering three-dimensional CT lateral cervical image showing the fusion of the C3-C4-C5 vertebral body (arrow) with partial posterior fusion of the C5-C6 vertebral body (arrowhead).

Initially, plain X-rays revealed a monobloc fusion of the C3-C4-C5 vertebral bodies with a partial posterior fusion of the C5-C6 vertebral bodies (Figure 2). Then, axial computed tomography (Figure 3) and three-dimensional reconstructions (Figure 4) confirmed the diagnosis and allowed measurement of the angles of the ‘S’-shaped cervicothoracic scoliosis and maximum axial vertebral rotation. 

In total, this cervical stiffness with other clinical and paraclinical signs falls within the framework of KFS. A family survey revealed no similar cases among relatives.

Since surgery was not indicated due to the risk of surgery and postoperative complications, our treatment was purely preventive and rehabilitative, based on stretching to maintain spinal flexibility. Aggressive manipulation was avoided due to the risk of spinal trauma. Posture correction to reduce strain on the cervical spine, and activity alternation to avoid prolonged postures were also recommended. Even with the patient's complete autonomy, daily activity adaptations, including ergonomic classroom and self-care modifications, were implemented to accommodate limited neck mobility.

Pain management included the use of muscle relaxants, benfotiamine (600 mg/day, administered in two divided doses for a duration of six months), nonsteroidal anti-inflammatory drugs (ketoprofen 50 mg was indicated for intense pain episodes, up to three times per day, but was rarely needed by the patient), and relaxing massages combined with gentle manual cervical traction, nighttime immobilization using a soft cervical collar, and alternating applications of heat and cold. With regular medical supervision, we prohibited activities that increase the risk of cervical trauma (team sports, therapeutic sports, therapeutic manipulation of the cervical spine). The patient also benefited from thoracic mobilization and respiratory muscle endurance training.

After one month, VAS decreased from 4/10 to 2/10, with improved posture and psychological well-being. Over two years of follow-up, the patient remained functionally independent without neurological deterioration or new complications, but without any change in joint RoM. The patient's mother, who accompanied the patient throughout all stages of treatment, played an important role in preventing traumatic complications, as did the application of self-programs at home and the importance of close medical follow-up. Prevention of secondary complications was also considered.

## Discussion

KFS occurs as a result of failure in normal segmentation of cervical mesodermal somites at two to eight weeks of gestation, but the cause is still unidentified [4]. The classical triad of the disease is: short neck, low posterior hairline, and decreased cervical joint RoM [5]. Although this triad constitutes the definition of KFS, recent publications have described many cases of KFS discovered incidentally during radiological assessments [5,8].

Cases of KFS can be accompanied by multiple anomalies. We wish to emphasize the importance of a thorough clinical examination, ideally conducted at an early age and before the onset of symptoms, as well as the need for appropriate adaptation measures [4,9]. In addition, several reports have described KFS with other congenital pathologies affecting the digestive system. Our patient has been under follow-up for hepatomegaly since the age of 12, with cystic dilatation of the subhepatic biliary tracts, secondary to Caroli’s disease. Although KFS associated with Caroli’s disease has not been previously reported in the literature, our case represents the first documented description of this association.

The fusion defect of KFS may be accompanied by scoliosis and/or kyphosis (60%), Sprengel deformity (30%), abnormalities of the urinary system (35%), deafness (30%), asymmetry (30%), facial asymmetry (20%), mirror movements (20%), and congenital heart disease (4.2e14%) [10]. Consistent with its higher prevalence in women, the syndrome was also observed in a female patient in our case.

Synkinesis, or mirror movement, is a voluntary movement on one side and an involuntary movement of the muscles on the contralateral side and is present in 20% of patients with KFS. This phenomenon is due to abnormal development of the corticospinal tract, its absence, or a lack of transcallosal inhibition. It is more common in patients with atlanto-occipital involvement, syringomyelia, or Chiari syndrome [11]. In our case, mirror movement was present in the upper limb, although the mentioned lesions were absent.

Although most patients with KFS are asymptomatic, they can develop additional symptoms such as axial neck pain and neurological dysfunctions (e.g., radiculopathy or myelopathy), which may remain latent until adulthood, as observed in our patient [4,12]. The variations in pathoanatomy and associated abnormalities in KFS necessitate a comprehensive evaluation, as treatment strategies can range from activity modification to extensive spinal surgery.

The prevalence of patients with KFS requiring surgery was 18.5%, with the majority undergoing posterior cervical surgery [13]. This is required in KFS, with basilar invagination, compression of the brain stem and upper cervical cord results in neurological deficits [14]. Participants who pursued surgery had more comorbidities and neurological symptoms but did not report more significant pain than non-surgically treated participants [4,13]. Prevention of various neck trauma, even minimal, remains the best way to avoid complications and is one of the pillars of current treatment, given the high risk of post-surgery complications and the inefficacy of rehabilitation means, which remain very limited [15]. 

Given its rarity, little research has been conducted to develop a suitable treatment plan for this pathology. A few articles have reported cases of KFS where management was based on physiotherapy and interventional techniques for pain management, such as ultrasound-guided hydrodissection of the cervical plexus nerves, the greater occipital nerve, and the suprascapular nerve, which provided pain relief [16]. In another case where they injected low doses of botulinum toxin (20 IU) into different muscles bilaterally, where a reduction in pain, stiffness, and improvement in posture was observed. However, the choice of muscles to be injected remained difficult [17]. Ultrasound-guided injections of the superficial cervical, suprascapular, and greater occipital plexus nerves relieved head, neck, and upper limb pain a few weeks later [16]. Manual cervical traction and muscle stretching of the neck and upper limbs help reduce pain and stiffness to improve mobility and posture. Therapeutic education was provided to master postural correction exercises, as well as breathing exercises to improve thoracic flexibility [18,19]. Medical pain management with level I and II analgesics, muscle relaxants, and gabapentin plays an important role in the treatment of KFS [16]. 

## Conclusions

With the increasing frequency of radiological assessments, KFS is no longer considered a rare disease. To date, there has been no consensus to codify the management of KFS. Given the progression of the disease, marked by complications and increased pain with age, and given the risk of complications related to surgical treatment, conservative rehabilitation can provide symptomatic relief and improve quality of life, though the RoM may remain unchanged. Interventional pain management techniques may be considered in selected cases, as supported by prior reports.
